# Non-destructive assessment of cannabis quality during drying process using hyperspectral imaging and machine learning

**DOI:** 10.3389/fpls.2024.1365298

**Published:** 2024-04-26

**Authors:** Hyo In Yoon, Su Hyeon Lee, Dahye Ryu, Hyelim Choi, Soo Hyun Park, Je Hyeong Jung, Ho-Youn Kim, Jung-Seok Yang

**Affiliations:** Smart Farm Research Center, Korea Institute of Science and Technology (KIST), Gangneung, Gangwon, Republic of Korea

**Keywords:** cannabidiol, classification, logistic regression, tetrahydrocannabinol, postharvest quality control

## Abstract

*Cannabis sativa* L. is an industrially valuable plant known for its cannabinoids, such as cannabidiol (CBD) and Δ9-tetrahydrocannabinol (THC), renowned for its therapeutic and psychoactive properties. Despite its significance, the cannabis industry has encountered difficulties in guaranteeing consistent product quality throughout the drying process. Hyperspectral imaging (HSI), combined with advanced machine learning technology, has been used to predict phytochemicals that presents a promising solution for maintaining cannabis quality control. We examined the dynamic changes in cannabinoid compositions under diverse drying conditions and developed a non-destructive method to appraise the quality of cannabis flowers using HSI and machine learning. Even when the relative weight and water content remained constant throughout the drying process, drying conditions significantly influenced the levels of CBD, THC, and their precursors. These results emphasize the importance of determining the exact drying endpoint. To develop HSI-based models for predicting cannabis quality indicators, including dryness, precursor conversion of CBD and THC, and CBD : THC ratio, we employed various spectral preprocessing methods and machine learning algorithms, including logistic regression (LR), support vector machine (SVM), k-nearest neighbor (KNN), random forest (RF), and Gaussian naïve Bayes (GNB). The LR model demonstrated the highest accuracy at 94.7–99.7% when used in conjunction with spectral pre-processing techniques such as multiplicative scatter correction (MSC) or Savitzky–Golay filter. We propose that the HSI-based model holds the potential to serve as a valuable tool for monitoring cannabinoid composition and determining optimal drying endpoint. This tool offers the means to achieve uniform cannabis quality and optimize the drying process in the industry.

## 1 Introduction

*Cannabis sativa* L. is a valuable industrial plant used as a raw material for producing various products including seed, oil, drugs, medicine, and fiber. Notably, cannabis plants contain cannabinoids such as cannabidiol (CBD) and Δ9-tetrahydrocannabinol (THC), which possess medicinal and psychoactive properties (Amin and Ali, 2019). In the cannabinoid biosynthesis pathway, both cannabidiolic acid (CBDA) and tetrahydrocannabinolic acid (THCA) serve as precursors for these active compounds. These acidic forms are synthesized from a single compound, cannabigerolic acid (CBGA), which are catalyzed by oxidocyclase enzymes (Tahir et al., 2021). Nonenzymatic thermal decarboxylation during heat exposure converts CBDA and THCA into their neutral forms CBD and THC, respectively. Typically, these end products are not present in growing cannabis but are typically formed through postharvest drying processes.

Drying is a crucial postharvest step in cannabis processing. Cannabis flowers contain approximately 80% water, and the drying process primarily prevents microbial growth and facilitates long-term storage (Lazarjani et al., 2021). Decarboxylation of cannabinoids is heat-induced; thus, the drying temperature and conditions, including humidity and pressure, are critical determinants affecting product quality (AL Ubeed et al., 2022). Turner and Mahlberg (1984) found that decarboxylation occurred when the leaves dried at 60°C, while at 37°C only cannabinoid acids were detected. According to Chen et al. (2021), hot-air drying increased CBDA conversion rate and decreased drying time as the temperature increased from 40°C to 90°C. The conventional drying method involves hanging and air drying in well-ventilated rooms, maintaining a temperature range of 18–21°C and a relative humidity of 50–55% (Challa et al., 2021). These conditions were designed to mitigate unwanted alterations in cannabis composition during the post-harvest process. However, current practices result in longer processing times, unnecessary expenses, and an increased risk of contamination (Das et al., 2022). Unfortunately, there are no established or predictive models for determining drying endpoints or total drying times. Moreover, even with identical drying conditions, dryness may vary considerably based on factors such as drying facility, flower size, and overall conditions. The adoption of real-time diagnosis technology for cannabinoid quality could potentially resolve issues related to drying endpoints and durations.

Hyperspectral imaging has emerged as a powerful tool for monitoring plant physiology and evaluating food quality in agriculture (Lu et al., 2020). Using the technologies, Jin et al. (2017) developed a model for predicting leaf water content, one of the important parameters for photosynthesis and biomass efficiency in *Miscanthus* plants. Jung et al. (2022) developed a diagnostic model for gray mold disease, including identification of asymptomatic infection sites on strawberry leaves. In particularly, the technologies for postharvest quality control have been developed, such as diagnosis of senescence status in broccoli plants during storage (Kabakeris et al., 2015), prediction of dietary fiber contents in fresh-cut celeries during storage (Yan et al., 2017), and prediction of anthocyanin content in purple sweet potato slices during drying process (Liu et al., 2017). In previous studies on cannabis, hyperspectral imaging has been utilized for plant identification (Pereira et al., 2020) and predict CBD and THC content (Lu et al., 2022).

The advantages of hyperspectral imaging, such as its speed, reliability, and non-destructiveness, broaden its potential use as a quality control technology for plant products (Kiani et al., 2018). However, extracting valuable information from high-dimensional data laden with redundant information and undesired noise owing to the measurement conditions is a significant challenge in hyperspectral image analysis (Saha and Manickavasagan, 2021). Therefore, the use of efficient algorithms and data-processing techniques is essential. Several spectral preprocessing techniques, including the Savitzky–Golay filter (SG filter), derivative (Der), and multiplicative scatter correction (MSC), have been utilized to address scattering, reduce noise, and enhance spectral features (Vidal and Amigo, 2012; Yoon et al., 2023). Machine learning algorithms provide an opportunity to establish classification or regression models that utilizes an extensive range of predictors in hyperspectral imaging, including logistic regression (LR), support vector machine (SVM), k-nearest neighbor (KNN), random forest (RF), and Gaussian naïve Bayes (GNB) (Saha and Manickavasagan, 2021).

Our study aims to confirm the hypothesis that CBD and THC concentrations change as drying progresses, regardless of consistent moisture content and weight. We developed a nondestructive method to evaluate the quality of cannabis flowers during drying using hyperspectral imaging and machine learning. To achieve this objective, we collected data on cannabinoid levels in flowers subjected to different drying conditions and durations. Several spectral preprocessing techniques, such as the SG filter, 1st Der, 2nd Der, and MSC, have been applied with several machine-learning algorithms, such as LR, SVM, KNN, RF, and GNB. The resulting model has the potential to monitor cannabis quality, optimize drying endpoints and duration, and enhance drying processes in the cannabis industry.

## 2 Materials and methods

### 2.1 Plant material and growth conditions

For this study, we utilized medical cannabis plants (*C. sativa* L.), specifically the ‘Cherry Blonde’ cultivar (Blue Forest Farms, NY, USA). The seeds were germinated in 40-mm peat pellets (Jiffy International, Kristiansand, Norway) using tap water in an indoor farming system. The growth conditions consisted of an air temperature of 24 ± 2°C/18 ± 2°C (day/night), relative humidity of 60 ± 5%, light intensity at a photosynthetic photon flux density (PPFD) of 200 µmol m^–2^ s^–1^, and 16-h photoperiod. After two weeks, the emerged seedlings were transferred to a cocopeat (chip: peat = 1:1) growbag (CocoGrow Cube 8.4 L, SJ Corp., Damyang, Korea). Irrigation was carried out using a drip irrigation system with a Hoagland nutrient solution. During the vegetative phase, all plants were grown under the identical conditions, except for the light intensity (PPFD of 400–450 µmol m^–2^ s^–1^) reaching the top of the plants. Cannabis flowers were induced by a short-day photoperiod, reducing light exposure from 16 h to 12 h during the reproductive phase. After 8 weeks of short-day conditions, we collected approximately 1 kg of fully matured female flowers for experimental and data-tracking purposes.

### 2.2 Drying conditions

Cannabis flowers were dried in six open plastic trays each under two drying and relative humidity conditions: hot-air drying (59 ± 3.6°C and 10 ± 3.7%) and cool-air drying (19 ± 1.2°C and 44 ± 8.2%). The cool-air drying conditions were similar to the traditional air-drying conditions (Challa et al., 2021). The air temperature and relative humidity were measured and recorded at 20-min intervals using a temperature and humidity data logger (RC-51H; Elitech Technology, Inc., Milpitas, CA, USA). The changes over time under these drying conditions are shown in **Supplementary Figure S1**. The flowers in each tray were weighed before drying and at 2, 4, 7, and 9 days post-drying. The relative weight change and relative water content (RWC) were calculated as follows:


$$\mathrm{Relative}\;\mathrm{weight}\;\mathrm{change}\;\left(\%\right)\;=\;{\text{w}}_{\text{t}}/{\text{w}}_{0}\times \;100$$

$$\mathrm{RWC}\;\left(\%\right)\;=\;\left({\text{w}}_{\text{t}}–\;{\text{w}}_{0}\times \;DM\right)/{\text{w}}_{t}\times \;100$$

where w*_t_* is the weight at time *t*, w_0_ is the fresh weight at harvest, and DM is the ratio of dry matter (DM = 0.21894) measured from flowers of the same cultivar. Flower samples were collected twice from five trays per treatment to obtain hyperspectral images and cannabinoid data for model development. For tracking data, weight and hyperspectral imaging data were collected from a single tray under each of the two drying conditions.

### 2.3 UHPLC analysis for cannabinoids

Flower samples were collected prior to drying and at 2, 4, 7, and 9 days after drying. Subsequently, the samples were promptly immersed in liquid nitrogen and were freeze-dried at –80°C. The lyophilized samples were ground finely, and the powder (1 g) was extracted using methanol (16 mL) under sonication at 50°C for 20 min. The extracts were centrifuged, filtered through a 0.22 µm membrane filter (Whatman, Maidstone, UK), and concentrated using a nitrogen gas evaporator. These concentrated extracts were then re-dissolved in DMSO to achieve a concentration of 10 mg/mL and stored at –80°C before analysis. The samples were diluted to 0.1 mg/mL with methanol before injection into an ultra-high-performance liquid chromatography (UHPLC) system. To quantify the target compounds, four standards (CBDA, CBD, THCA, and THC) were purchased from Cerilliant (Cerilliant Corp., Round Rock, TX, USA) and dissolved in acetonitrile at a concentration of 1 mg/mL. UHPLC analysis was performed using a Shimadzu Nexera X3 UHPLC system (Shimadzu Corp., Kyoto, Japan), equipped with two pumps (LC-40B), a column oven (CTO-40C), an autosampler (SIL-40C), and a photodiode array (SPD-M40). Separations were achieved on a YMC-Triart C18 column (100 × 2.0 mm, 1.9 µm; YMC Co., Ltd., Kyoto, Japan), with a mobile phase composed of 0.2% formic acid in both water (A) and acetonitrile (B). The gradient elution was set as follows: 75% B for 0–4 min, linear increase to 90% B in 4–10 min, decreased to 75% B within the next 0.5 min and re-equilibrated to initial gradient 75% B until 13 min. The column temperature was 30°C. The sample injection volume was 5 µL.

### 2.4 Hyperspectral image collection and processing

**Figure 1:**
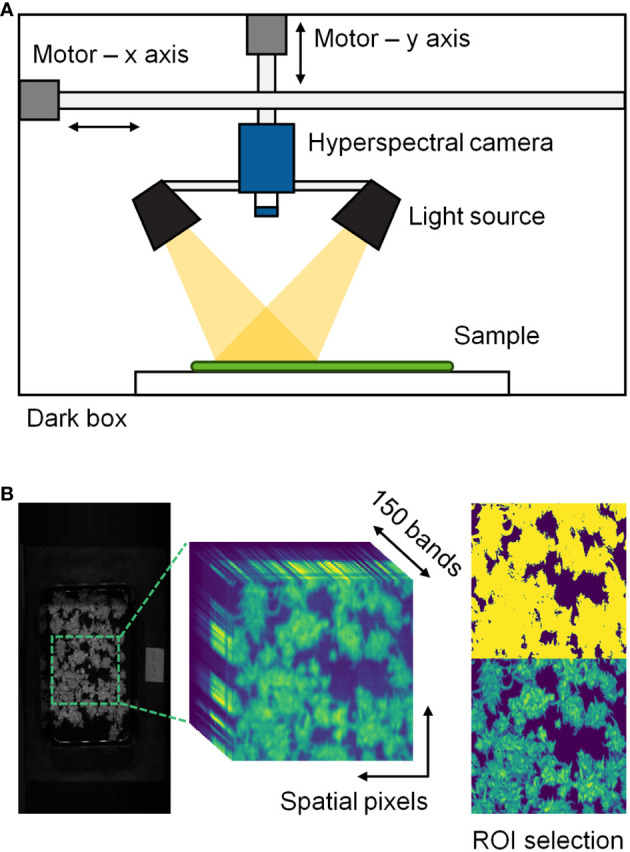
Hyperspectral imaging system **(A)** and description of hyperspectral data processing **(B)** in this study. The regions of interest (ROI) were selected through a threshold technique.

The hyperspectral imaging system consisted of a hyperspectral imaging camera (MicroHSI 410 SHARK, Corning, NY, USA) and eight 20 W halogen lamps placed within a movable stage in a dark chamber (**Figure 1**). The camera captured line-scan images with 150 spectral bands in the 400–1,000 nm range at a rate of 100 mm/s. The hyperspectral images were obtained at a spatial resolution of 682 × 1,540 pixels. Each round of scanning involved five samples for the experimental data and one tray for the tracking data. Hyperspectral data were examined within Python 3.9 environment (Python Software Foundation, Wilmington, DE, USA) using the Spectral Python (SPy) library.

A threshold technique was used to eliminate the background from the hyperspectral cube data. A normalized band difference (NBD) was calculated using reflectance values at 764.74 and 684.69 nm, where NBD = (R_764.74_ – R_684.69_)/(R_764.74_ + R_684.69_) and R denotes the reflectance values at the wavelength in a single pixel. A threshold was applied to the images to enhance the contrast between the plants and background, and pixels with NBD > 0.3 were selected as regions of interest (ROI) (**Figure 1B**). Classification data were collected by extracting multiple ROI from 90 hyperspectral images. Only regions with 20 × 20 pixels covering more than 70% of the ROI were selected without overlapping were selected, resulting in 27–73 data points for each sample. An average spectrum was extracted from each data point, and a total of 4,707 spectral data were used to develop the classification model.

### 2.5 Model development for quality classification

Four quality characteristics, namely dryness, CBDA conversion, THCA conversion, and CBD : THC, were categorized into two or three classes based on RWC and cannabinoid content (**Table 1**). To determine the classes of each characteristic, we conducted a sensitivity analysis for each range using the raw spectrum and a logistic regression model (**Supplementary Figure S2**).

**Table 1:** Classification class for quality of cannabis flowers during drying process.

Quality	Class		Number of data		
			Total	Calibration	Prediction
Dryness	Extreme dried	0< RWC ≤ 10	2,202	1,761	441
	Dried	10< RWC ≤ 40	1,839	1,471	368
	Fresh	40< RWC ≤ 100	666	533	133
CBDA conversion	Low CBD%	0< C/TC ≤ 20	2,505	2,004	501
	Middle CBD%	20< C/TC ≤ 60	1,001	801	200
	High CBD%	60< C/TC ≤ 100	1,201	960	241
THCA conversion	Low THC%	0< T/TT ≤ 20	2,594	2,075	519
	Middle THC%	20< T/TT ≤ 50	1,631	1,305	326
	High THC%	50< T/TT ≤ 100	482	385	97
CBD : THC	High C:T	0< C/T< 20	1,371	1,097	274
	Extreme high C:T	20 ≤ C/T ≤ 100	3,336	2,668	668

RWC, relative water content; C/TC, CBD percentage of total CBD (CBD + CBDA); T/TT, THC percentage of total THC (THC + THCA); C/T, ratio of total CBD to total THC.

To predict and classify the quality traits of harvested cannabis flowers, we compared combinations of different spectral preprocessing methods and machine learning algorithms. Five spectral preprocessing methods were used: raw spectrum, SG filter with a third-order polynomial fit with five data points: 1st Der, 2nd Der, and MSC. The pre-processed average spectra are shown in **Supplementary Figure S3**. Five machine learning algorithm classifiers were used: LR, SVM, KNN, RF, and GNB. We applied the one-vs.-rest method to binary classification algorithms, such as LR and SVM, for multiclass classification. The model implementation was programmed in Python 3.9 based on SciPy and the scikit-learn package.

### 2.6 Model evaluation and statistical analysis

For model development and evaluation, the dataset was randomly divided into calibration and prediction sets in a ratio of 8:2. The calibration set was used to train the models and determine the spectral preprocessing method and classifier based on the highest accuracy using 10-fold cross- validation. The final model was subsequently evaluated using the prediction set, and the results were presented as a confusion matrix and receiver operating characteristic (ROC) curve. The four evaluation metrics were computed from the confusion matrix values, as follows:


Accuracy = (TP + TN)/(TP + TN + FP + FN)

Precision = TP/(TP + FP)

Recall = TP/(TP + FN)

F1-score = 2 × (Precision) × (Recall)/(Precision + Recall)

where TP is true positive; FP, false positive; TN, true negative; and FN, false negative. Accuracy is the ratio of correct estimates to all predictions, and precision is the ratio of correct estimates to all positive predictions. The F1-score is defined as the harmonic average of recall and precision, indicating the overall accuracy of the classification. The ROC curve represents the changes in the true positive rate (recall) and false positive rate by the threshold. The area under the curve (AUC_ROC_) was calculated from the ROC curve to evaluate the predictive performance of the models. To confirm the applicability of the final model for monitoring, we tested it on tracking data.

The weight and cannabinoid content of the cannabis flowers were compared using two-way ANOVA and Tukey’s honestly significant difference (HSD) test to assess the effects of the drying method and period. Statistical analyses were performed using R software (R 4.2.2; R Foundation, Vienna, Austria).

## 3 Results

### 3.1 Changes in weight and water content of cannabis flowers during drying process

**Figure 2:**
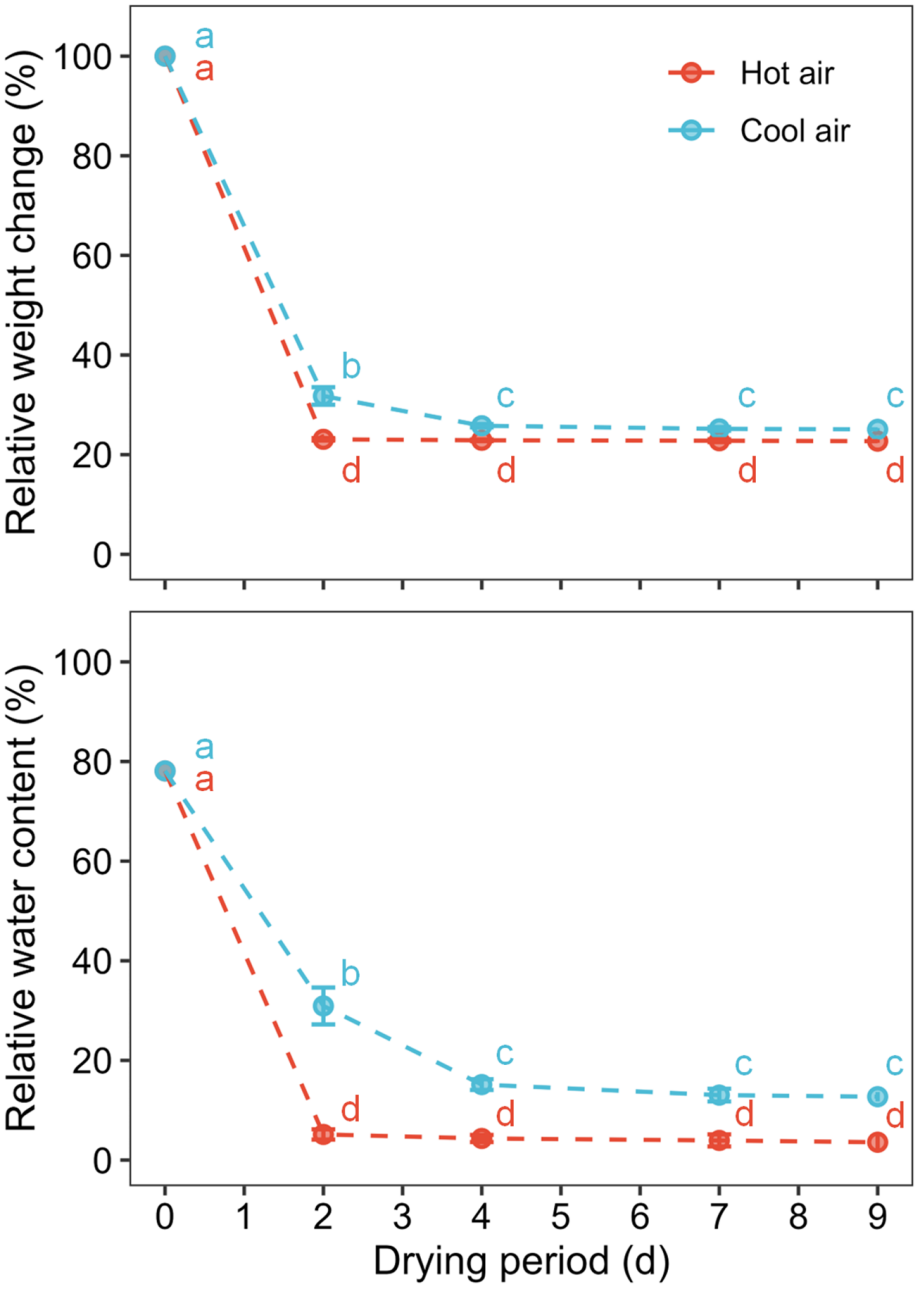
Relative weight change and water content of cannabis flowers during the drying period according to drying method: hot-air and cool-air drying. Circle and vertical bars indicate mean ± SD (n = 6). Different letters indicate significant differences among drying method and period at *p*< 0.05 by two-way ANOVA and Tukey’s HSD test.

The relative weight and water content of the cannabis flowers decreased rapidly during the initial two days of drying under both hot- and cool-air conditions (**Figure 2**). In particular, flowers subjected to hot-air conditions were completely dried after two days, with no significant changes observed in weight and RWC. During the nine days of the drying period, RWC declined from 78.1% to 12.7 ± 0.9% under cool-air conditions and 3.6 ± 1.4% under hot-air conditions.

### 3.2 Spectrum and color changes of cannabis flowers during drying process

**Figure 3:**
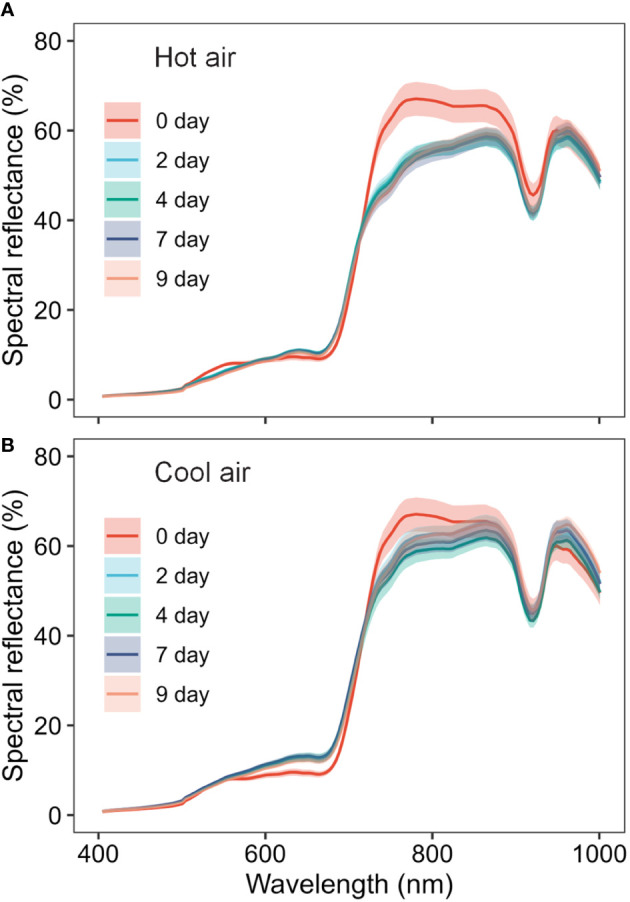
Spectral changes of cannabis flowers during the drying period (0, 2, 4, 7, and 9 days) according to drying method (**A**, hot-air drying; **B**, cool-air drying). Solid lines and shaded areas indicate mean ± SD (n = 10).

**Figure 4:**
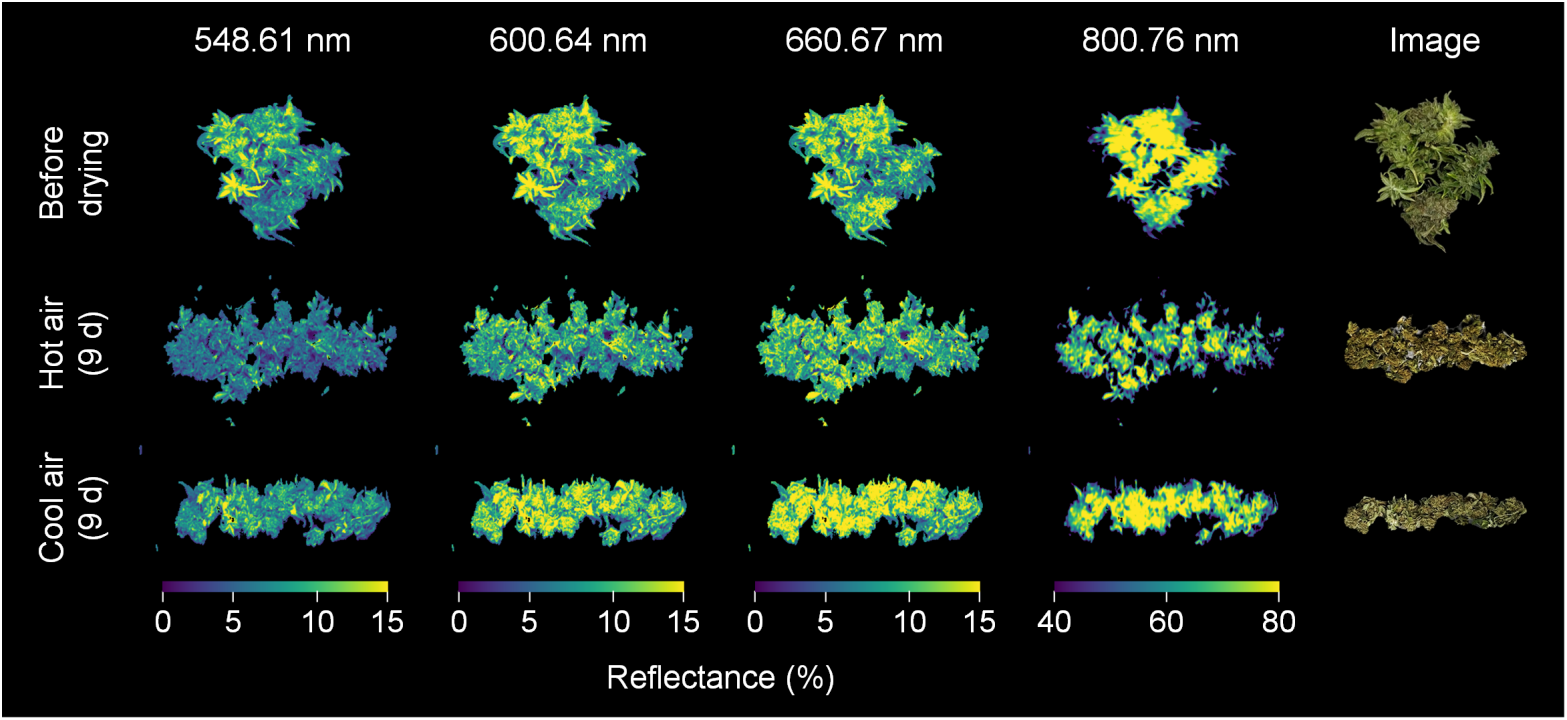
Spectral images of cannabis flowers before and after 9 days of drying by hot-air and cool-air drying. Color indicates spectral reflectance at 548.61, 600.64, 660.67, and 800.76 nm, respectively.

The average spectra of the hyperspectral images revealed the spectral reflectance of cannabis flowers, and the variations were more closely associated with the drying method than to the drying duration (**Figure 3**). The drying-induced changes in reflectance were categorized into four ranges, and representative spectral images are shown in **Figure 4**. The reflectance at wavelengths below 552.61 nm was lower after hot-air drying compared to other drying conditions. In contrast, higher values were observed in the range of 556.61–612.65 nm after cool-air drying in comparison to alternative conditions. For spectra within the wavelength range of 616.65–708.7 nm, the highest values were observed after cool-air drying, followed by hot-air drying, with the lowest levels prior to drying. Notably, reflectance in the range of 720.71–884.81 nm experienced a rapid decrease under both drying conditions, with the most pronounced decline observed after only two days of hot-air drying. Conversely, cool-air drying resulted in gradual decrease over a longer drying period.

### 3.3 Changes in cannabinoids of cannabis flowers during drying process

**Figure 5:**
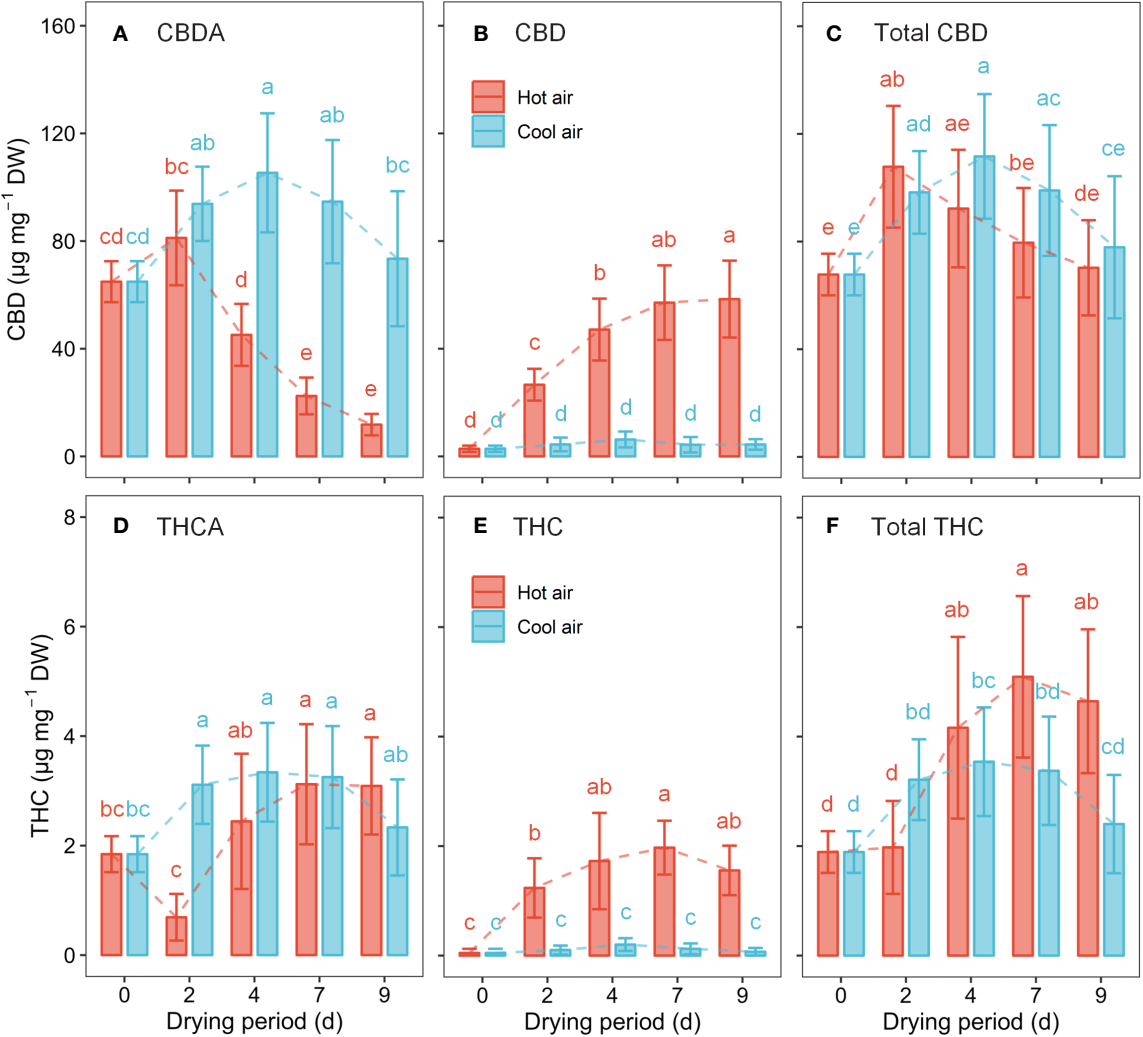
Cannabinoid concentration in cannabis flowers during the drying period according to hot-air and cool-air drying methods: CBDA **(A)**, CBD **(B)**, total CBD **(C)**, THCA **(D)**, THC **(E)**, and total THC **(F)**. Bars and vertical bars indicate mean ± SD (n = 10). Different letters indicate significant differences among drying method and period at *p*< 0.05 by two-way ANOVA and Tukey’s HSD test.

Cannabinoids, such as CBDA, CBD, THCA, and THC, underwent significant changes during the drying process (**Figure 5**). The results of the two-way ANOVA revealed that the drying method, drying period, and their interaction considerably influenced cannabinoid content (*p*< 0.01), except for total CBD. Total CBD concentration was significantly affected by the duration of drying, whereas the drying method (*p* = 0.065) and their interaction (*p* = 0.098) were not significant. Under hot-air conditions, CBDA gradually declined between days 4–9 of drying, resulting in a 65.4% decrease at day 7 compared to the initial value. However, the CBD content rapidly increased after hot-air drying, surging by a 20.1-fold after 7 days of drying compared to the initial value. The total CBD content peaked at 48 h post hot-air drying, with a 59.1% increase from the initial value. After 7 days of hot-air drying, THCA, THC, and total THC concentrations reached their highest levels, increasing by 1.7-, 41.1-, and 2.7-fold, respectively, compared to their pre-drying levels. Meanwhile, under cool-air conditions, CBDA and THCA reached their maximum values after 4 days of exposure, showing increases of 62.3% and 81.2%, respectively, compared with their levels before drying. However, no significant differences were observed in CBD and TCH levels during the cool-air drying period. After 4 days of exposure to cool-air drying, the total CBD and total THC levels increased by 64.8% and 87.2%, respectively, compared to their initial levels before drying.

### 3.4 Cannabis quality assessment models based on hyperspectral imaging

**Figure 6:**
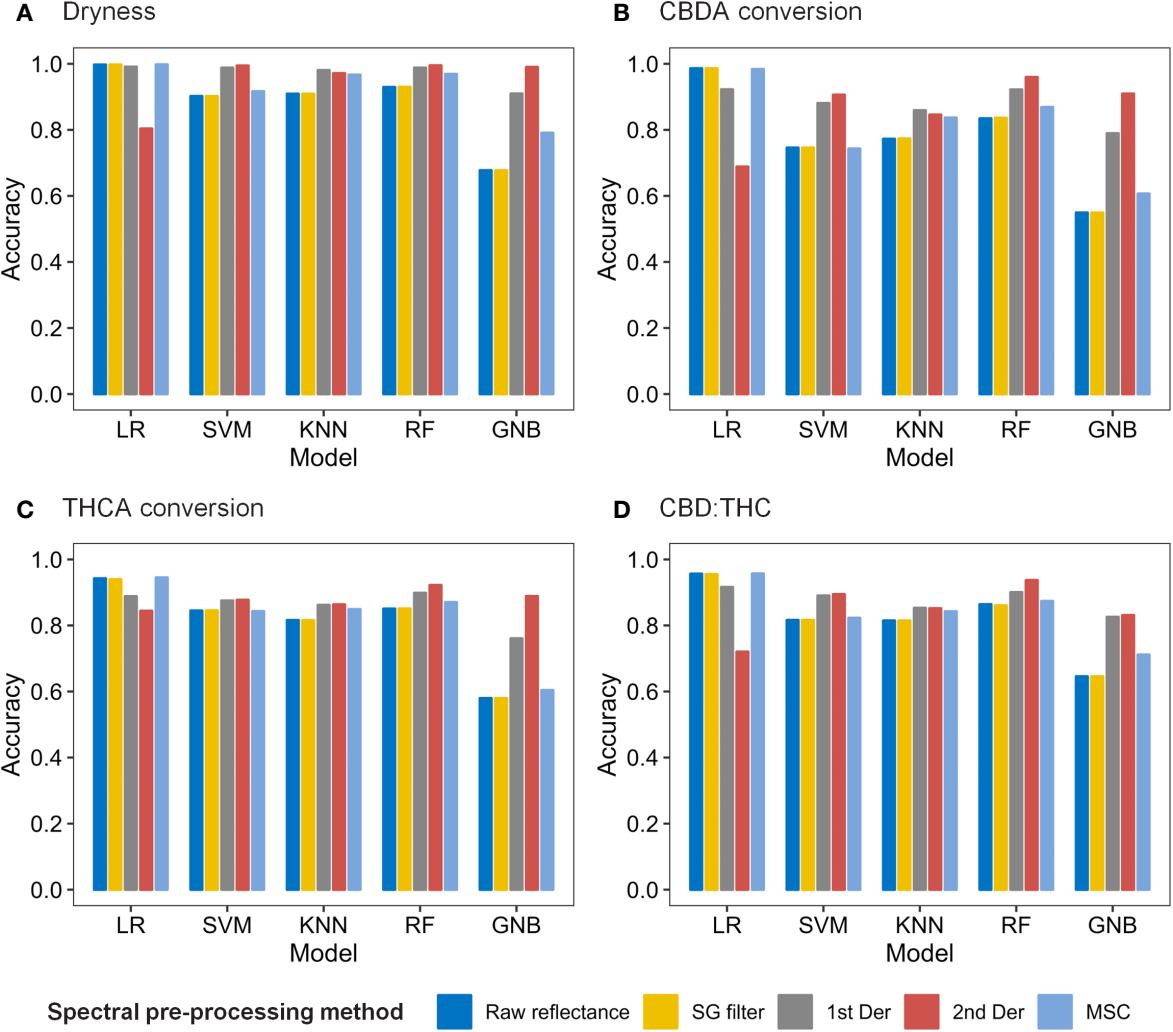
Accuracy of 10-fold cross validation results for dryness **(A)**, CBDA conversion **(B)**, THCA conversion **(C)**, and CBD : THC **(D)** according to spectral data pre-processing methods and machine learning models.

As a result of the 10-fold cross-validation (CV), the spectral preprocessing method and machine learning model were determined for each quality characteristic (**Figure 6**). The LR model had the highest overall accuracy when coupled with the MSC, SG filter, or raw reflectance, followed by the RF model with 2nd Der. Regarding the classification of dryness, THCA conversion, and CBD : THC, the LR model with MSC preprocessing achieved the highest 10-fold CV accuracies of 0.9979, 0.9450, and 0.9570, respectively. To classify the CBDA conversion, the LR model with the SG filter method was selected owing to its CV accuracy of 0.9862.

The selected models were evaluated using five metrics, and they demonstrated high accuracy in predicting each quality characteristic (**Table 2**). The prediction accuracy closely aligned with the CV accuracy of each model. All confusion matrices and ROC curves used to calculate these metrics are detailed in the (**Supplementary Figures S4**, **S5**). Only the THCA conversion model exhibited precision, recall, and F1-score values lower than the accuracy values, indicating an imbalance among the classes. Among the THCA conversion classes, the high THC% class contained a relatively small amount of data (10% of all data), which consequently led to lower precision, recall, and F1-score values (**Table 1**, **Supplementary Figure S4**).

**Table 2:** Prediction performance of the selected model for cannabis quality in the test set.

Quality	Spectral pre- processing	Model	Prediction performance				
			Accuracy (%)	Precision (%)	Recall (%)	F1-score (%)	AUC_ROC_
Dryness	MSC	LR	99.7	99.7	99.6	99.7	1.00
CBDA conversion	SG filter	LR	98.1	97.2	97.4	97.3	1.00
THCA conversion	MSC	LR	94.7	89.9	89.5	89.7	0.99
CBD : THC	MSC	LR	95.8	95.0	94.6	94.8	0.99

AUC_ROC_, area under ROC curve.

### 3.5 Application for cannabis quality assessment during drying process

**Figure 7:**
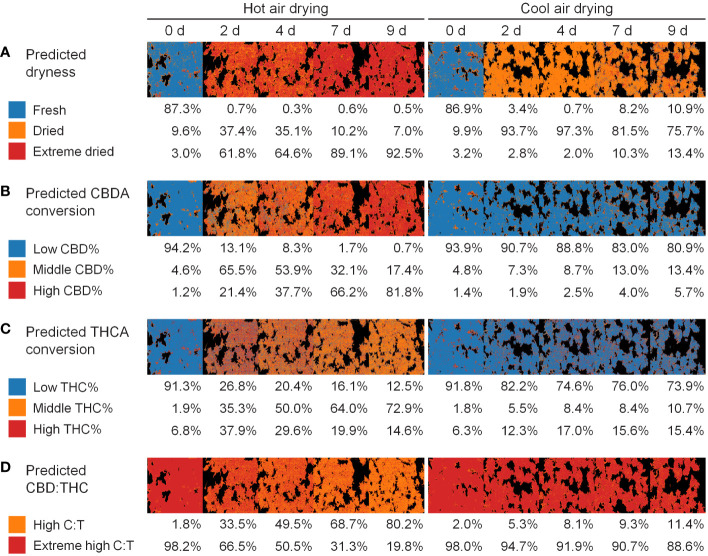
Application for quality monitoring of cannabis flowers in drying method and period: dryness **(A)**, CBDA conversion **(B)**, THCA conversion **(C)**, and CBD THC **(D)**. Colors represents the predicted class. Percentage values indicate the proportion of the class occupied by plant pixels.

The dryness level did not significantly change following hot-air drying, whereas other quality aspects, particularly the CBDA conversion, and CBD : THC ratio, showed variations (**Figure 7**). A prediction model based on hyperspectral imaging can be extended from single-pixel-level classification to visualize the distribution of each class. The developed models were used to monitor the cannabis quality during the drying process. This model facilitated the continuous tracking of cannabis quality through changes in compounds during the drying process.

## 4 Discussion

We investigated the changes in cannabis quality during the drying process and devised a nondestructive method for evaluating the quality of cannabis flowers using hyperspectral images with machine learning algorithms. Although the weight and RWC remained constant during the drying process, the concentrations of CBD, THC, and their precursors varied depending upon the drying conditions. Therefore, cannabis quality is inevitably determined by drying endpoints and conditions.

### 4.1 Changes in cannabinoid composition in cannabis plants

Major cannabinoids, including CBDA, CBD, THCA, and THC, share a biosynthetic pathway that connects to the precursor molecule, CBGA (Tahir et al., 2021; Govindarajan et al., 2023). This biosynthesis predominantly occurs within the trichomes of cannabis plants (Livingston et al., 2020; Tanney et al., 2021), which develop in various parts of female cannabis flowers. While trichome development may vary among different genotypes, it typically begins gradually after the onset of flowering, with a significant increase observed at approximately 3–4 weeks as female flowers take form. As the trichomes continue to mature, they synthesize and accumulate cannabinoids. However, the senescence phase begins at approximately 8 weeks after flower anthesis, and resin secretion gradually decreases (Punja et al., 2023).

Cannabinoid trichomes can be categorized into four types: non-secretory, sessile capitate, pre-stocked capitate, and stocked-capitate trichomes (Hammond and Mahlberg, 1973). The heads of the stocked-capitate trichomes are protected by a cuticle layer. Within the lower part of these trichome heads, 12–16 disc cells can be found where cannabinoid synthesis occurs (Hammond and Mahlberg, 1973; Small and Naraine, 2016; Livingston et al., 2020). In contrast, the upper part of the resin accumulated various secondary metabolites, including cannabinoids, terpenes, organic acids, and polysaccharides (Jin et al., 2020; Livingston et al., 2021; Tanney et al., 2021). Cannabidiolic acid synthase (CBDAS) and Δ9-tetrahydrocannabinolic acid synthase (THCAS), responsible for the synthesis of THCA and CBDA, respectively, from CBGA, are equipped with secretory signal peptides that guide them to the resin. CBDAS and THCAS, once secreted into the extracellular space, catalyze the conversion of CBGA to Δ9-THCA and CBDA (Taura et al., 2007).

Remarkably, trichomes maintain their physical integrity even after the drying process owing to the protective cuticle layer covering their heads. This preservation of trichome heads suggests that no spatial limitations hinder the catalytic activity of THCAS and CBDAS during the drying period (Taura et al., 2007; Meija et al., 2022). This study provides limited evidence to support the preserved functional capacity of cannabinoid synthesis (**Figure 5**). Further research is needed to understand the relationship between trichome preservation and precursor turnover during drying.

Cannabinoid acids, such as CBDA and THCA, are readily decarboxylated and stabilized by heat during the drying process (Tahir et al., 2021; Govindarajan et al., 2023). Excessive heat can lead to the loss of the synthesized cannabinoids. Although the drying temperatures applied in this experiment were not high enough to cause significant cannabinoid loss (Wang et al., 2016; Das et al., 2022), prolonged drying can cause such loss (Addo et al., 2021; Chen et al., 2021). Therefore, determining the drying endpoint supports a smooth and stable transition to the next step.

### 4.2 Cannabis quality in industrial processes

The industrial decarboxylation process is crucial for extracting the active components, CBD and THC, through heating at a relatively high temperature, approximately 100°C, for a short reaction duration (Wang et al., 2016). In this study, CBDA conversion gradually increased with longer drying times, reaching 83.2% after 9 days of hot-air drying (**Figure 5**). CBD chemotype plants with a CBD : THC ratio of approximately 25:1 are commonly used in medical-grade products (Chandra et al., 2017). The ‘Cherry Blonde’ cultivar used in this study is a CBD chemotype cannabis with low THC levels, with a CBD : THC ratio of 36:1 at harvest. Under hot-air conditions, the ratio increased to 55:1 at 2 days after drying and subsequently decreased to 22–15:1 at 4–9 days after drying (**Figure 5**). The THCA conversion followed a similar pattern, peaking at 62.5% 2 days after hot-air drying and subsequently decreasing to 41.5–33.5% during days 4–9. In contrast, under cool air conditions, the conversion rates of CBDA and THCA and the CBD : THC ratio remained below 6% and in the range 29–36:1, respectively. These findings suggest that cool air can effectively maintain the conversion rates and desired ratios. Although additional decarboxylation process is required for medical-grade cannabis production, preservation is a suitable postharvest strategy, particularly for extended transportation and storage, ensuring a longer shelf life (AL Ubeed et al., 2022).

Each class of the four qualities in the present study was determined as the range that could be best classified through sensitivity analysis (**Supplementary Figure S2**). A criterion for classifying the industrial quality of cannabis is required to accelerate the development of quality control technologies.

### 4.3 Hyperspectral imaging analysis with spectral pre-processing and machine learning

We established a hyperspectral imaging-based model for evaluating cannabis quality during the drying process, including dryness, CBDA conversion, THCA conversion, and CBD : THC ratio. Extracting valuable information from hyperspectral data is challenging because of high dimensionality, redundancy, and noise (Saha and Manickavasagan, 2021). To make hyperspectral imaging applications more adaptable for real-time use, efficient algorithms and data processing techniques are necessary.

The most common practices of spectral preprocessing used in this study were derived from chemometric techniques, including the SG filter, 1st and 2nd derivatives, and MSC (**Supplementary Figure S3**). The SG filter is one of the most well-known smoothing methods for denoising, such as instrumental noise or extreme band rejection. It is also used to interpolate spectral data that can be used for other transformations, such as derivatives. In particular, the hyperspectral imaging of plants requires additional correction techniques because of the variability arising from these complex geometries (Mishra et al., 2017). Derivative techniques effectively reduce the additive effects as a constant offset and linear baseline shift. These techniques not only emphasize spectral features but also increase noise levels in the data (Vidal and Amigo, 2012). MSC is widely used to compensate for additive or multiplicative effects, i.e., both light scattering and baseline shift corrections. In this study, preprocessing methods, except for the SG filter, significantly affected the accuracy of the models (**Figure 6**, **Supplementary Figure S3**). The similarity in model accuracy between the SG filter and the raw spectrum may be attributed to the stable conditions in the dark chamber, which indicate minimal noise from the measurement environment (**Figure 1**).

Among the various machine-learning algorithms, we used common supervised classification methods, including LR, SVM, KNN, RF, and GNB. In this study, LR models exhibited the highest accuracy in predicting the four qualities when coupled with the MSC or SG filters (**Figure 6**). LR is a probability-based algorithm based on a logistic (sigmoid) function that calculates the probability of a binary outcome (Saha and Manickavasagan, 2021). The probability (*P*) with multiple variables has the following general form:


$$P=\;1/\left(1+e^{-\left(\beta_{0}+\beta_{1}X_{1}+\beta_{2}X_{2}+\ldots +\beta_{n}X_{n}\right)}\right)$$

where *X*_1_ to *X*_n_ are distinct independent variables; *β*_0_ to *β*_n_ are the regression coefficients. When the number of samples or predictors is limited, e.g., in the field of clinical prediction models (Christodoulou et al., 2019; Nusinovici et al., 2020), LR is considered more suitable than other machine learning models. However, the predictor variables in the hyperspectral image-based model were wavelength bands, and the number was not small (150 in this study). Because of the assumption of a linear relationship between the features and class labels, LR may not effectively capture complex nonlinear relationships. For instance, LR models have limitations in inferring relationships between gene expressions in large-scale profiling (Chen et al., 2016). In this study, the accuracy of the LR model was low only when combined with the 2nd Der pre-processing, likely due to the increased feature complexity (**Figure 6**). In contrast, the accuracy of the SVM, KNN, RF, and GNB models increased when combined with 1st or 2nd derivatives compared with other preprocessing methods (**Figure 6**). These models are appropriate for handling non-linear relationships. SVM is an algorithm that determines the hyperplane that maximizes the distance between different classes in the data. It was designed to resolve overfitting issues when dealing with high-dimensional data (Noble, 2006). KNN is an instance-based algorithm that assigns data to the major class among its *k* nearest neighbors, where *k* = 5 in this study (Rehman et al., 2019). RF is a bagging algorithm that constructs multiple decision trees and combines their predictions (Breiman, 2001). In previous studies using hyperspectral data, SVM and RF models were more accurate than the KNN model in predicting nitrogen accumulation in legume plants (Flynn et al., 2023). GNB uses Bayes’ theorem with the assumption of feature independence and the Gaussian distribution of each class and classifies data based on probability (Frank et al., 2000). These results might be attributed to the linear relationship between spectral features and cannabis quality, or because the preprocessing makes the data more linear or removes nonlinear variations that better match the assumption of LR.

### 4.4 Industrial quality assessment techniques based on hyperspectral imaging

Non-destructive analytical technique using spectroscopy, include Fourier transform infrared (FT-IR), near-infrared (NIR), Raman spectroscopy, and hyperspectral imaging (Xu et al., 2023), ensure rapid and accurate analysis, and qualitative and quantitative evaluation. In addition to its advantages, hyperspectral imaging allows the extension of spectral analysis results of one pixel to a spatial distribution level. If there is no priority among the qualities, the spatial homogeneity of the components can be evaluated using the coefficient of variance (Yoon et al., 2022). For cannabis products, the quality priority depends on the purpose of the drying process. Traditional drying methods, such as the cool-air conditions used in this study, aim to reduce disease or damage and increase shelf life (Challa et al., 2021). As the conversion rates of CBDA and THCA or the CBD : THC ratios were preserved under these conditions, it would be more appropriate to monitor the quality based on dryness or absolute content (**Figure 7A**). Although the cannabinoid content can vary depending on the cannabis cultivar or developmental characteristics, the accuracy of the binary classification was high (**Supplementary Figure S6**). Therefore, caution should be exercised when using the cannabinoid content as a criterion. The classification model for the total CBD content was predicted with an accuracy of 0.741 by the RF model combined with 2nd Der, and the model for the total THC content was predicted with an accuracy of 0.833 by the LR model without spectral preprocessing. The thresholds for defining CBD and THC levels were 90 µg mg^−1^ and 3 µg mg^−1^, respectively, according to a previous study (Lu et al., 2022). Consequently, the developed model for dryness- or variety-specific cannabinoid content based on hyperspectral imaging can serve as a supporting technology to reduce unnecessary time and enhance quality control for conventional and large-scale drying processes. Conversely, industrial decarboxylation process is an essential step for extracting the active components, CBD and THC, through heating at a relatively high temperature around 100°C and a short reaction time (Wang et al., 2016). The endpoint of drying was determined when the samples with a high CBDA conversion rate occupied more than 80% of the image, corresponding to 9 days of hot-air drying (**Figure 7B**). Accordingly, the developed models for the conversion rate or ratio of cannabinoids enable the monitoring of cannabis quality and determination of the drying endpoint, regardless of the nonuniform environment within the drying facility, contributing to optimizing the industrial drying process.

## 5 Conclusion

Our study analyzed the dynamic factors affecting cannabis quality during the drying process and introduced a nondestructive quality assessment approach using hyperspectral imaging and machine learning. Despite the constant weight and water content throughout the drying process, the cannabinoid content varied with drying conditions. Thus, our findings emphasize the importance of determining a precise drying endpoint to maintain consistent cannabinoid levels. Drying processes can be performed for two different purposes: to preserve cannabinoid composition at relatively low temperature or to induce decarboxylation of the acid form through heat treatment. Both purposes require monitoring techniques for uniform quality, which can be accurately predicted through the integration of hyperspectral imaging and machine learning techniques.

The results of this study indicate that the hyperspectral imaging model can be used as a valuable tool for monitoring the quality of cannabis in industrial products. This tool not only facilitates the evaluation of the uniformity of cannabis quality but also aids in the identification of the optimal drying endpoint, even in the context of large-scale and non-uniform conditions. We anticipate that our findings will catalyze future investigations aimed at improving drying processes, and thereby contributing to the advancement of the cannabis industry and the development of cutting-edge quality control technologies.

## Data availability statement

The raw data supporting the conclusions of this article will be made available by the authors, without undue reservation.

## Author contributions

HY: Conceptualization, Data curation, Formal analysis, Investigation, Methodology, Software, Validation, Visualization, Writing – original draft, Writing – review & editing. SL: Conceptualization, Data curation, Formal analysis, Investigation, Methodology, Resources, Writing – original draft. DR: Data curation, Formal analysis, Investigation, Methodology, Writing – original draft. HC: Investigation, Resources, Writing – original draft. SP: Methodology, Software, Validation, Writing – review & editing. JJ: Funding acquisition, Resources, Writing – review & editing. H-YK: Formal analysis, Methodology, Writing – review & editing. J-SY: Conceptualization, Project administration, Supervision, Writing – review & editing.
