# Supplementary material for: Non-destructive assessment of cannabis quality during drying process using hyperspectral imaging and machine learning
Source: Front Plant Sci. 2024 Apr 26;15:1365298. doi: 10.3389/fpls.2024.1365298 (PMC11082398; doi:10.3389/fpls.2024.1365298)
Supplement: Supplementary file 1 [file DataSheet_1.docx]

Supplementary Material


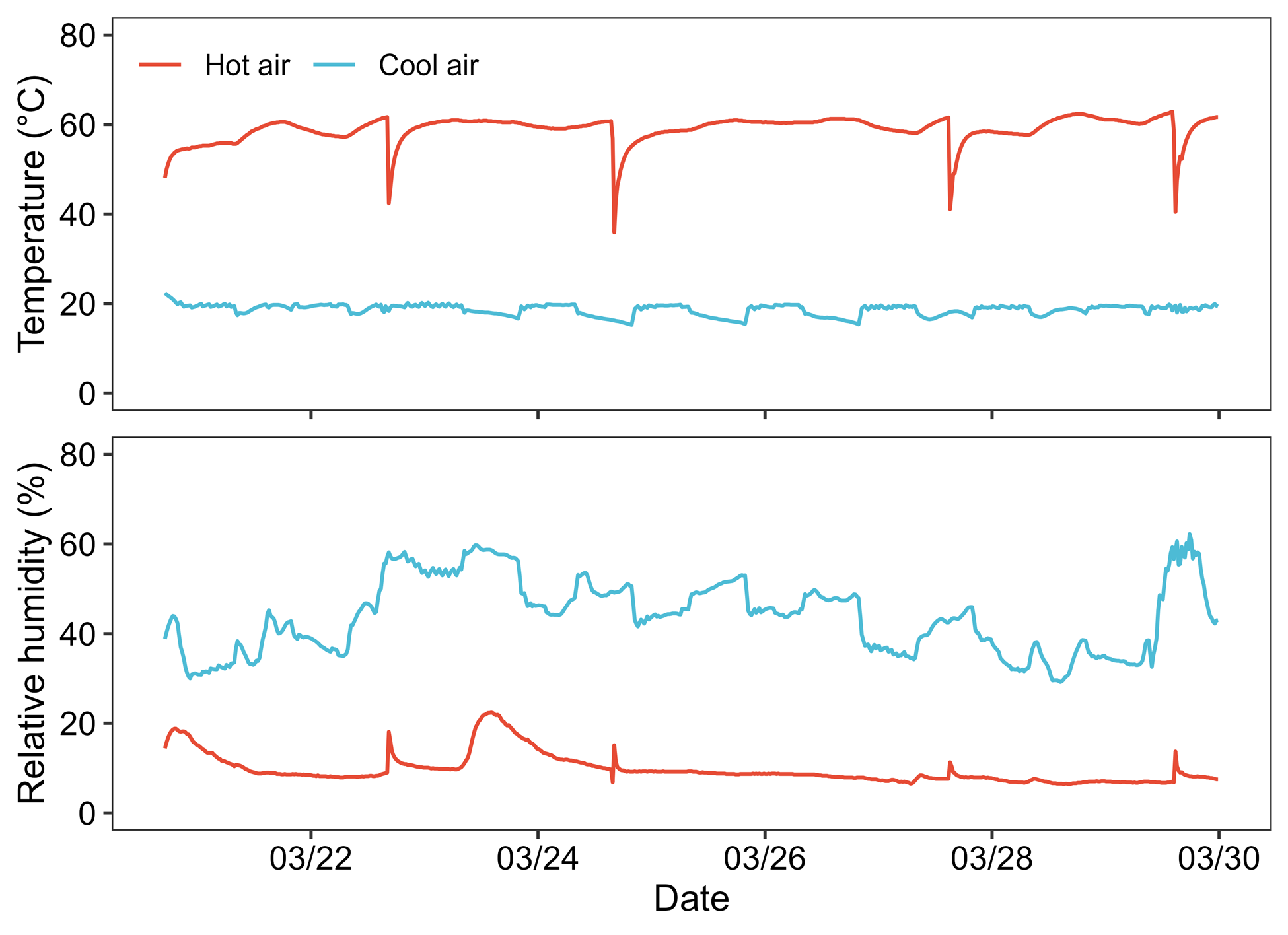


**Supplementary Figure S1.** Changes in air temperature and relative humidity under hot-air and cool-air drying conditions.

**
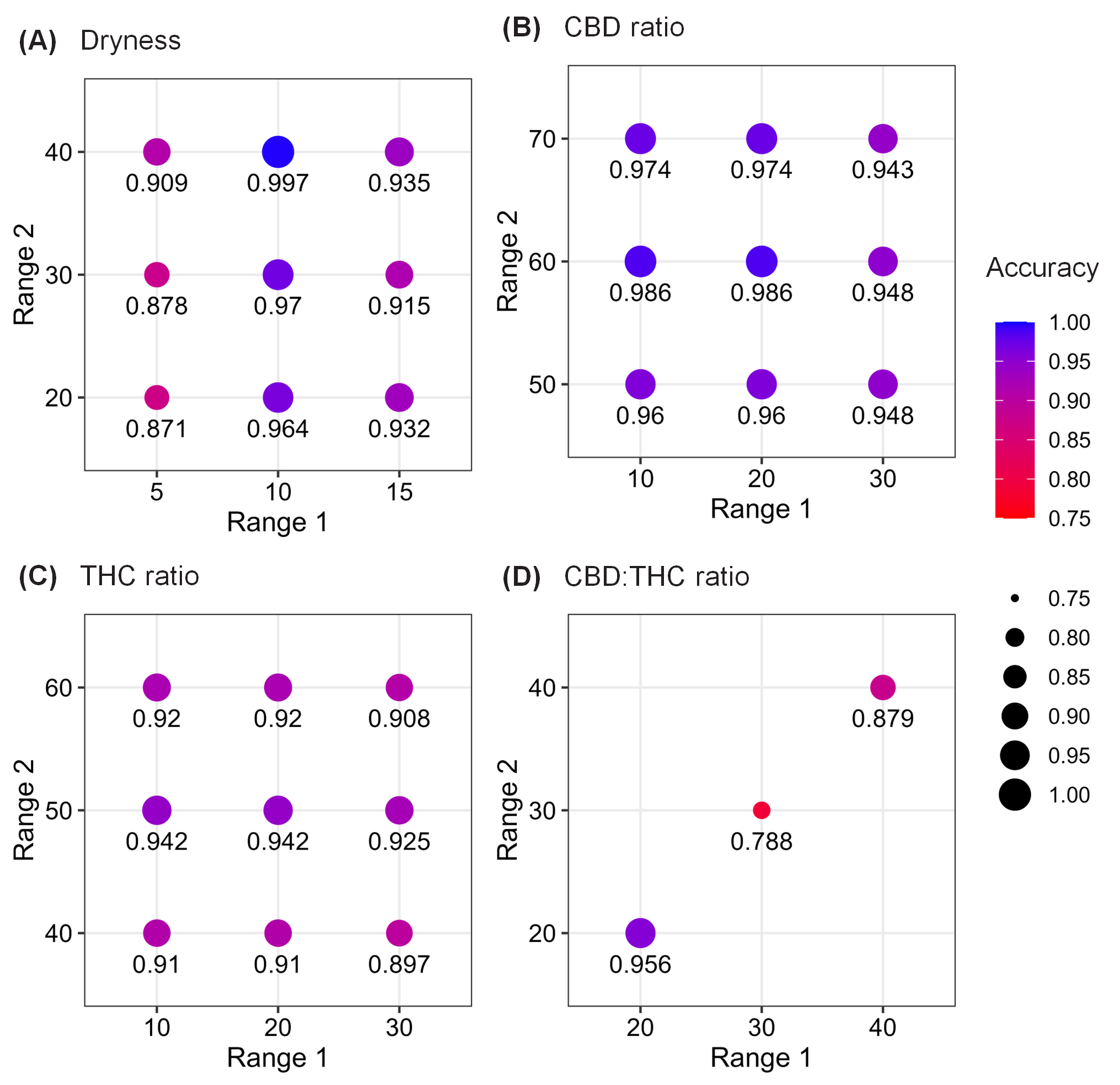
**

**Supplementary Figure S2.** Sensitivity analysis result: accuracy for classifying cannabis quality with each range of classes using raw spectrum and logistic regression model. Range 1 and 2 represent the threshold dividing each quality into 2-3 classes.


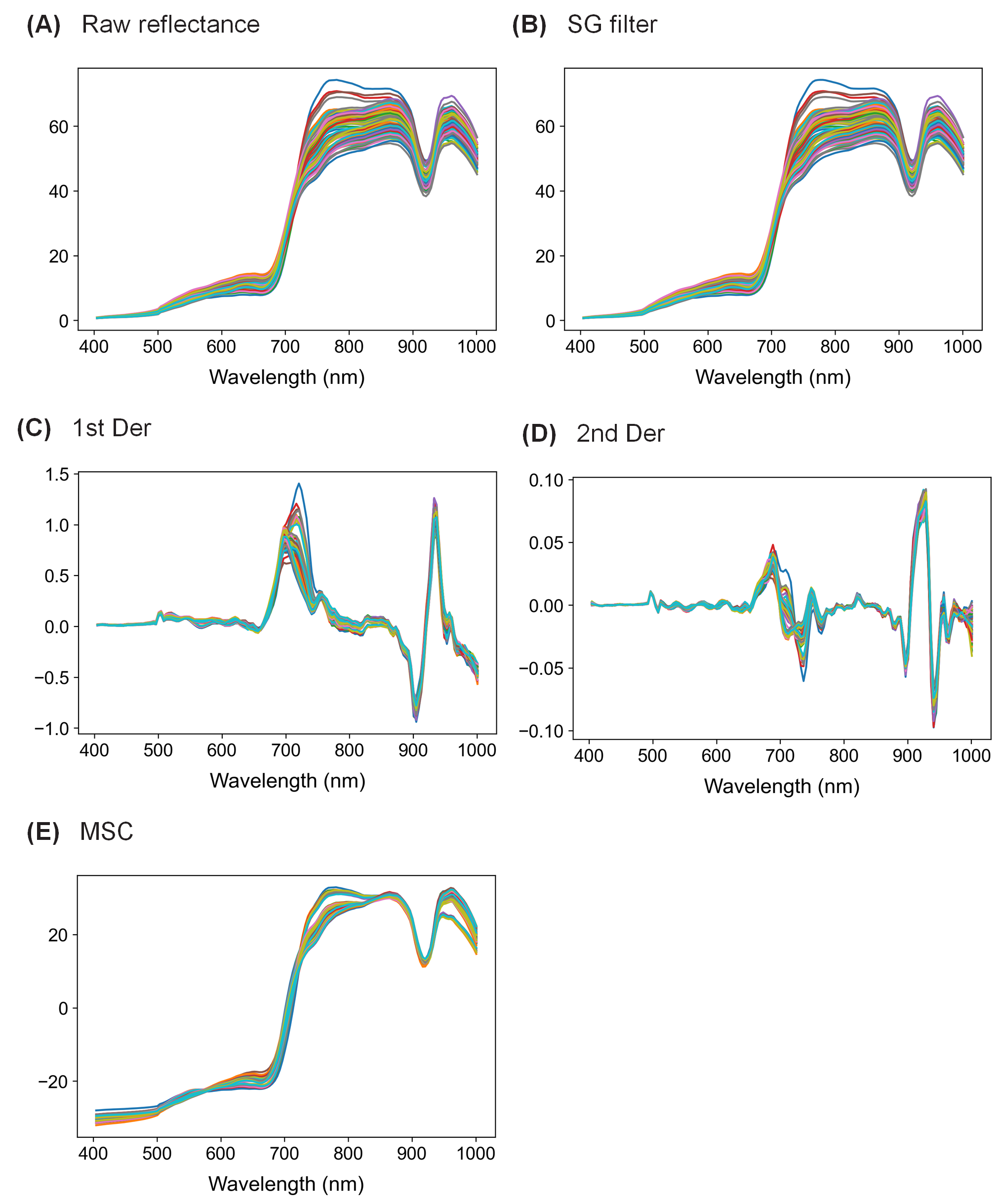


**Supplementary Figure S3.** Raw reflectance (A) and pre-processed spectral data (B-E) of cannabis plants. The spectral pre-processing methods used were Savitzky–Golay filter (SG filter; B), 1st and 2nd derivative after SG filter (1st Der and 2nd Der), and multiplicative scatter correction (MSC). Different color lines indicate mean spectra of different drying methods and periods.


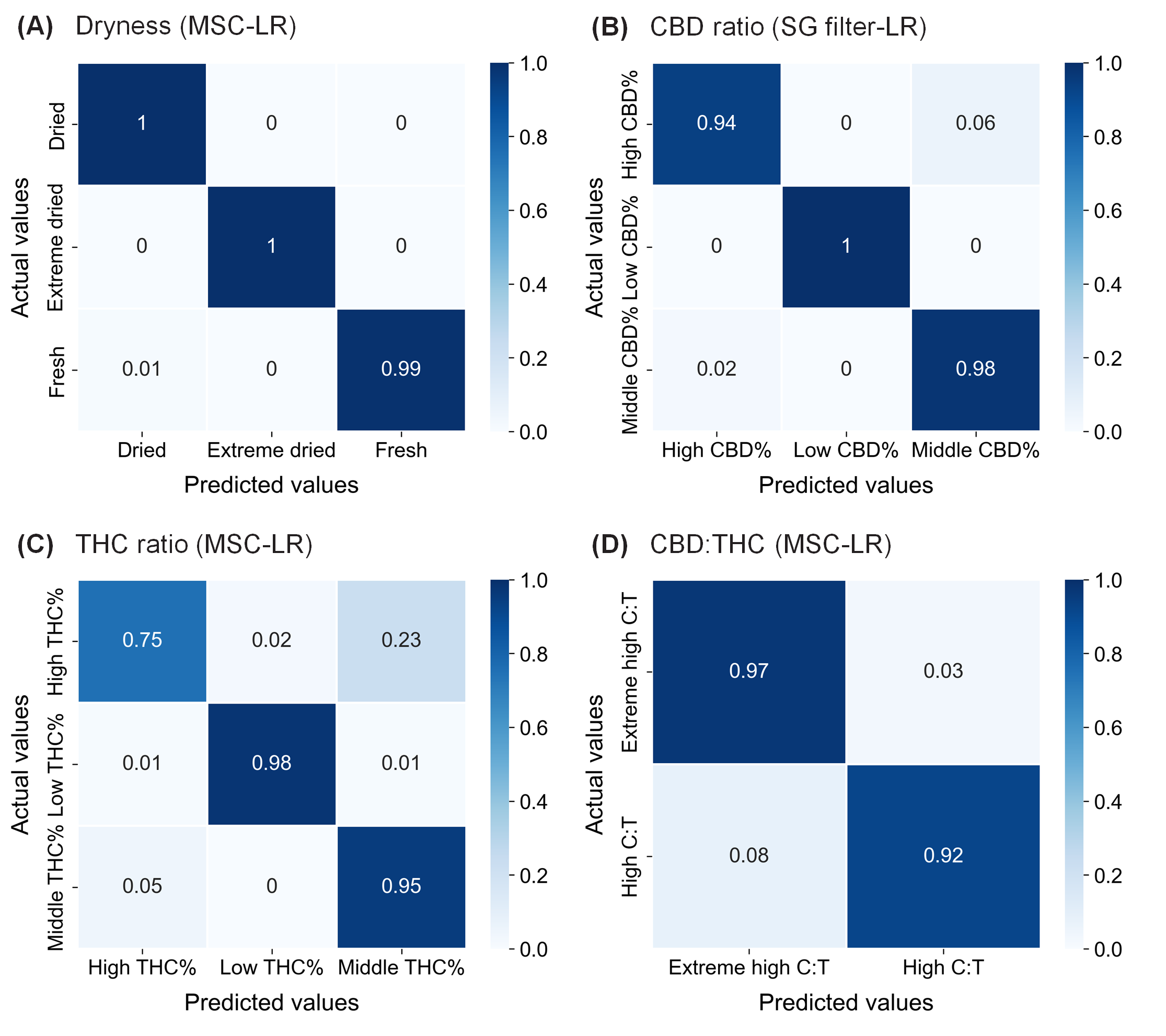


**Supplementary Figure S4.** Normalized confusion matrix of the selected models for dryness (A), CBDA conversion (B), THCA conversion (C), and CBD:THC ratio (D).


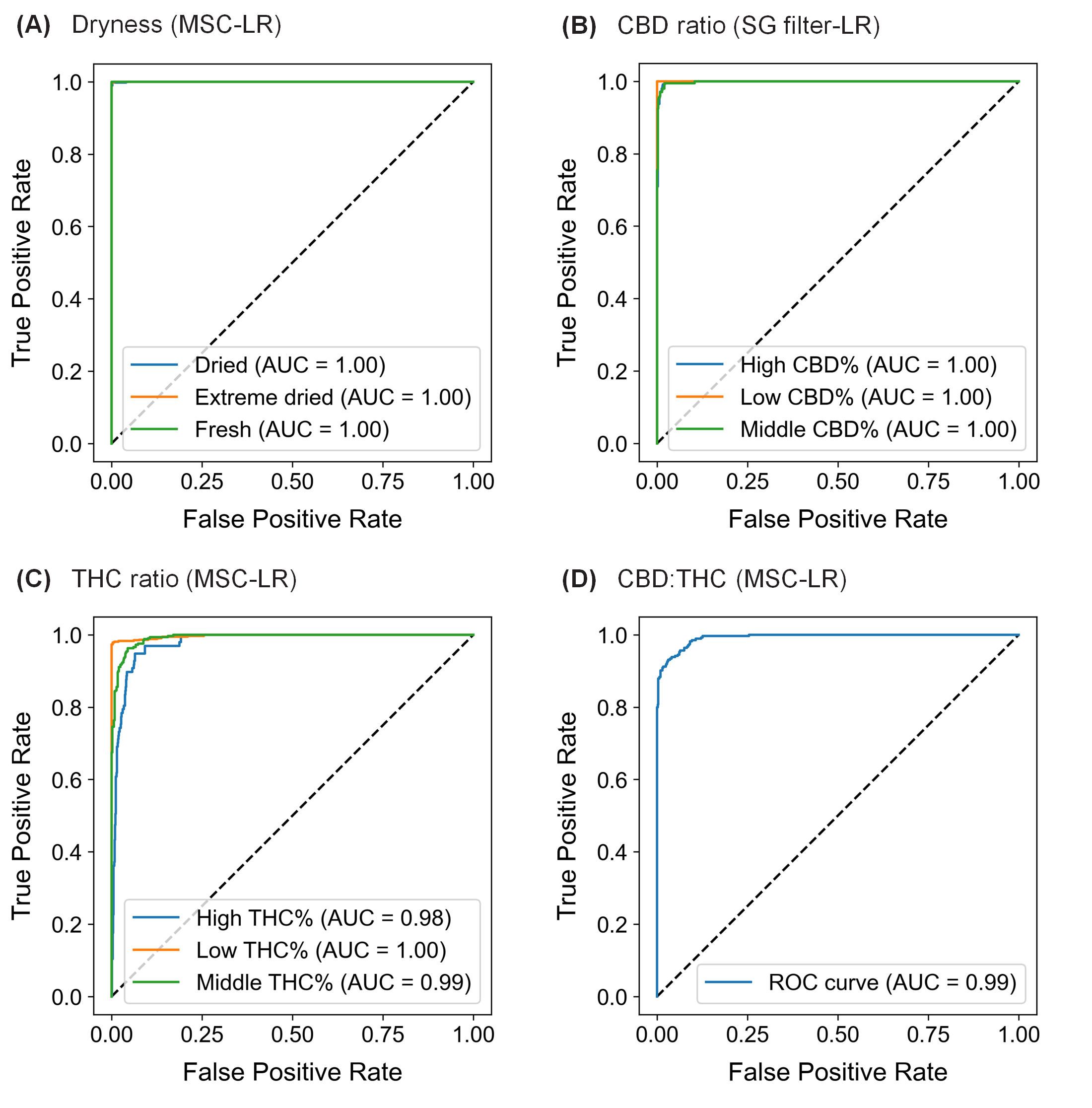


**Supplementary Figure S5.** ROC curve of the selected models for dryness (A), CBDA conversion (B), THCA conversion (C), and CBD:THC ratio (D). AUC, area under each curve.


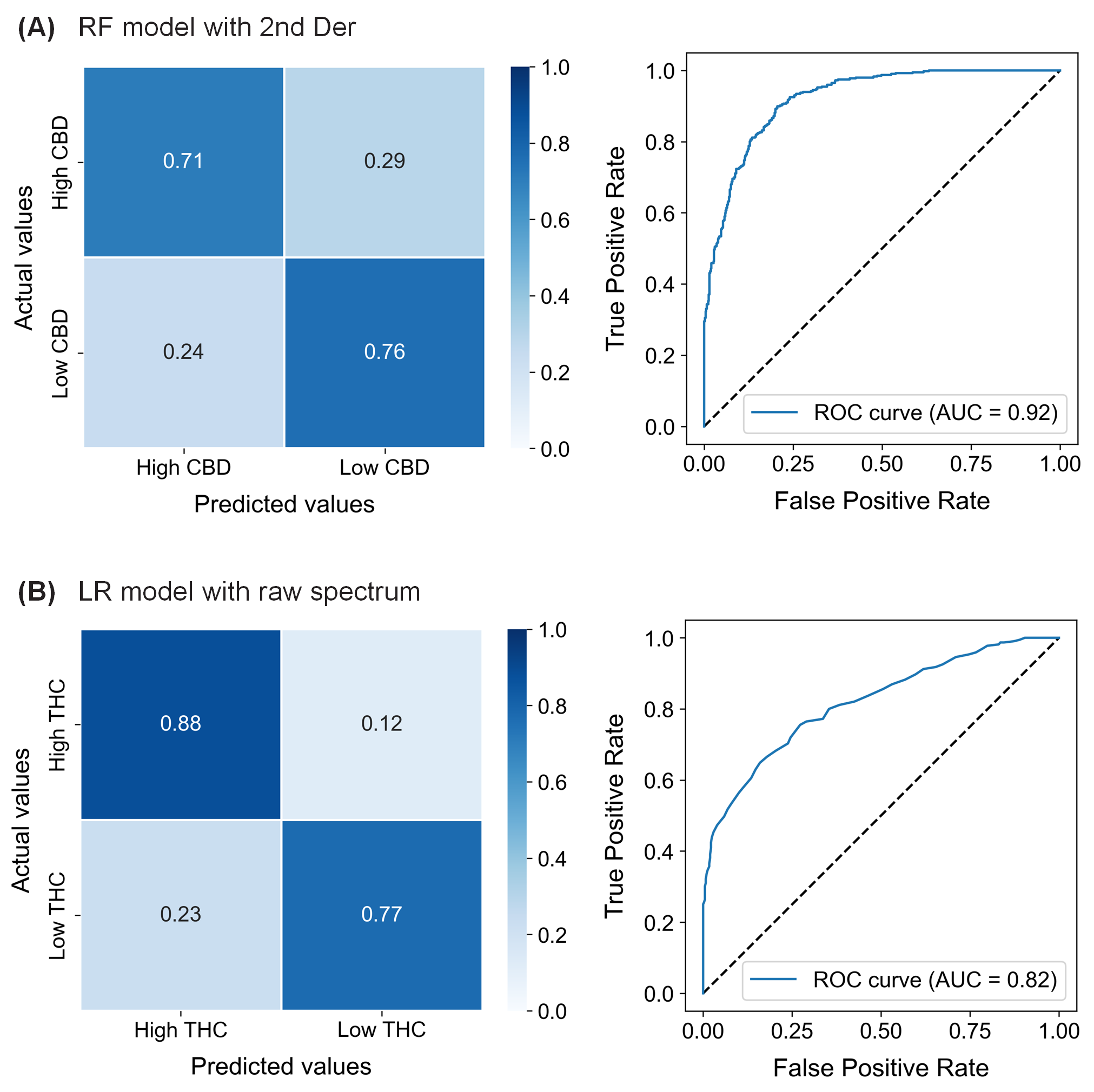


**Supplementary Figure S6.** Prediction performance in the test set: Confusion matrix and ROC curve from the best model for total CBD content (A), and total THC content (B). AUC, area under curve. CBD levels (high and low) were defined based on 90 µg mg^−1^ DW, and THC levels were defined based on 3 µg mg^−1^ DW.
